# The Influence of Halloysite Clay on the Properties of the Polybutylene Succinate (PBS)/Sawdust, PBS/Sugarcane Bagasse, and PBS/Sawdust/Sugarcane Bagasse Hybrid Composites

**DOI:** 10.3390/polym17152120

**Published:** 2025-07-31

**Authors:** Tlholohelo Sylvia Sikhosana, Ntsoaki Joyce Malebo, Mpho Phillip Motloung, Tladi Gideon Mofokeng, Mokgaotsa Jonas Mochane

**Affiliations:** 1 Department of Life Sciences, Central University of Technology, Bloemfontein 9301, South Africa; 220036826@stud.cut.ac.za (T.S.S.); nmalebo@cut.ac.za (N.J.M.); 2 Department of Chemistry, College of Science, Engineering, and Technology, University of South Africa, Johannesburg 1709, South Africa; motlomp1@unisa.ac.za; 3 Centre for Nanostructures and Advanced Materials, DSI-CSIR Nanotechnology Innovation Centre, Council for Scientific and Industrial Research, Pretoria 0001, South Africa; tmofokeng@csir.co.za

**Keywords:** polybutylene succinate, natural fibres, expandable graphite, hybrid composite, halloysite

## Abstract

In this study, the influences of natural fibres (sugarcane bagasse (SB) and sawdust (SD)) on the material properties of polybutylene succinate (PBS) prepared through melt compounding were investigated. The study further evaluated the effects of incorporating halloysite nanotubes (HS) and expandable graphite (EG) on the properties of PBS/SD and PBS/SB binary and PBS/SB/SD hybrid composites. The morphological analysis indicated poor interfacial adhesion between PBS and the fibres. The obtained findings indicated enhancements in the complex viscosity of PBS in the presence of natural fibres, and further improvements in the presence of HS and EG. The stiffness of PBS hybrid composites also increased upon the addition of HS and EG. Moreover, the crystallization temperatures of PBS increased in the presence of fillers, with EG showing better nucleation efficiency. However, the mechanical properties (toughness and impact resilience) decreased due to the increased stiffness of the composites and the poor interfacial adhesion between the matrix and the fillers, indicating the need to pre-treat the fibres to enhance compatibility. Overall, the material properties of PBS/SD/SB hybrid composites were enhanced by incorporating HS and EG at low concentrations.

## 1. Introduction

In recent years, biodegradable polymers have been regarded as suitable replacements for conventional plastics due to their biodegradability and potential to alleviate the plastic pollution caused by conventional polymers such as polyolefins [1,2]. Among various existing biodegradable polymers, poly (butylene succinate) (PBS) has received considerable attention due to its appealing properties including good processability, biodegradability, and thermal and chemical resistance. Nevertheless, PBS has shortcomings that limit its applications, such as softness, a limited Young’s modulus, low viscosity and melt strength, and availability at exorbitant prices [3,4,5,6]. To mitigate these drawbacks, the development of sustainable PBS composites comprising low-cost materials such as natural fibres has emerged due to their unique advantages such as availability at low to zero cost, abundance, high specific modulus, biodegradability, and environmental friendliness [4,7,8]. Natural fibre-reinforced biodegradable polymer composites are an interesting class of materials that are eco-friendly, cost-effective, and biodegradable [9,10].

Hybrid composites comprising two or more natural fibres are even more fascinating due to the possibility of obtaining a more diverse range of properties than those that are attained by single-fibre-reinforced composites. Furthermore, hybrid bionanocomposites consisting of natural fibres and nanomaterials have also generated interest due to a combination of properties resulting from the fillers’ sizes, ranging from micro- to nano-scale, and the high surface area of nanomaterials [7,8]. The incorporation of nanomaterials into natural fibre-reinforced composites has proven effective in enhancing the properties of polymer/natural fibre composites. Haris et al. [8] comprehensively reviewed some of the natural fibre/polymer hybrid composites containing at least one type of nanomaterial. Their summarized studies displayed enhancements in the material properties of natural fibre/polymer composites in the presence of nanoparticles. However, it is worth mentioning that there is a lack of studies in the literature on PBS/natural fibre hybrid bionanocomposites. Mochane et al. [4] alluded to the necessity of also diverting research attention to PBS/natural fibre hybrid composites, as a way of widening the applications of PBS/natural fibre composites and addressing the challenges associated with PBS.

A few studies have been reported on PBS/natural fibre hybrid bionanocomposites. The studies attempted to address some of the challenges associated with PBS and PBS/natural fibre composites. Most recently, Theys et al. [11] prepared PBS/maize stalk fibre hybrid bionanocomposites containing modified montmorillonite (MMT) nanoclay and expandable graphite (EG). Their findings indicated a better improvement in the storage modulus and flame retardancy of the PBS/maize stalk fibre in the presence of modified MMT, which acted as a compatibilizer compared to EG. Horiuchi et al. [6] fabricated hybrid bionanocomposites of PBS/canabrava fibres/lignin containing both MMT and sepiolite (SEP) clays. The authors reported enhancements in the crystallization temperature (T_c_), thermal stability, and stiffness of the PBS/canabrava fibres/lignin, with SEP showing better improvements compared to MMT. Motloung et al. [12] noticed an increase in the melt viscosity and accelerated degradation of PBS in the presence of chitin fibres and hydroxyapatite nanoparticles. These findings substantiate the need to explore the use of other natural fibres in developing PBS-based hybrid composites with enhanced properties, as this would broaden the application scope of PBS and widen the choice of natural fibres for a specific property improvement.

Natural fibres such as sugarcane bagasse (SB) and sawdust (SD) are lignocellulosic waste products with the potential to develop sustainable PBS composites. SD is a natural fibre obtained as a waste product of wood working and is mostly used for animal bedding, mulch, etc. [13,14]. SB is a fibrous lignocellulosic waste material obtained after the crushing and extraction of juice from sugarcane stalks [15,16]. Both SD and SB fibres have been employed in the fabrication of PBS composites. Frollini et al. [17] and Feng et al. [18] evaluated the properties of PBS reinforced with SB. On the other hand, a few studies on PBS/SD composites have been reported. Hongsriphan et al. [19] and Petchwattana et al. [20] investigated the influence of accelerated weathering on the properties of PBS/SD composites. In the current study, both SD and SB were used as a reinforcing agent in PBS as a viable way to re-purpose and add value to them. In addition, these materials are available at low to zero costs. Thus, they can contribute significantly to the reduction of PBS costs, which are usually reflected in the product costs. In South Africa, about 7 million tons of SB is produced annually, whereas about 4.7 million m^3^ of SD is generated in South Africa [21,22]. This demonstrates the necessity to utilize these abundantly available lignocellulosic waste products in polymer composites.

To our knowledge, PBS-based hybrid composites comprising both SB and SD and/or combining either of these fibres with nanomaterials have not yet been reported. Nanomaterials such as halloysite nanotubes (HS) hold great potential for the enhancement of the properties of polymer–natural fibre-reinforced composites. HS is a naturally occurring nanoclay that exhibits intriguing properties such as a high surface area, biocompatibility, and low toxicity, and it can be integrated with other materials in composites to enhance their properties [23,24]. Hasan et al. [25] developed jute fibre-reinforced poly (3-hydroxy-butyrate-co-3-valerate) (PHBV)-reinforced composites containing HS. A significant increase in the thermal and mechanical properties of PHBV/jute fibre composites was noticed when adding HS. In addition, fillers with layered structures such as (EG) can be used to further improve the properties of natural fibre-reinforced polymer composites. EG is widely used in polymer composites to impart important properties including thermal and electrical conductivity, thermal stability, flame retardancy, and improved stiffness [26,27]. Theys et al. [11] incorporated EG into PBS/maize stalk composites and noticed improvements in flame retardancy and thermal stability compared to PBS/maize stalk composites.

Due to the limited research on PBS-based hybrid composites, it is imperative to investigate the influences of different filler combinations (hybrid systems) on the properties of PBS. This will shed light on the specific types of fillers that can be used to attain certain properties for a specific application. Therefore, in this study, the influences of natural fibres (SD and SB) in combination with HS and EG on the material properties of PBS are investigated. To the best of our knowledge, the effects of the simultaneous incorporation of SB and SD on the properties of PBS are currently uncertain. In addition, hybrid composites based on SD and SB as well as HS and EG have not yet been explored. Therefore, the current study seeks to expand and contribute to the existing knowledge on PBS/natural fibre hybrid composites containing these fillers, with the aim of addressing the challenges associated with PBS, while also beneficiating low-cost and underutilized natural fibres such as SD and SB fibres as reinforcing agents in green PBS composites. The incorporation of both EG and HS can yield a synergistic enhancement of the properties of natural fibre-filled PBS composites containing SD and SB due to their intrinsic properties, such as their high surface area and capacity to act as nucleating and reinforcing agents in polymer composites. Thus, the study intends to develop novel PBS-based hybrid composites based on SD and SB in the presence of HS and EG. The study further explores the influence of HS on the material properties of PBS/SD, PBS/SB, and PBS/SD/SB hybrid composites, and that of EG on the properties of PBS/SD/SB/HS hybrid composites.

## 2. Materials and Methods

### 2.1. Materials

The polymer used in this study, polybutylene succinate (PBS), was acquired from 2 MBIO-Engineering Polymers, Ramanathapuram, India, in the form of pellets. It exhibited a melt flow index (MFI) of 2.16 kg/10 min at 190 °C, a heat combustion of 23.6 KJ/g, a melting temperature of about 120 °C, and a density of 1.25 g/cm^3^. Ground sawdust (SD) was acquired from a timber processing plant in Bloemfontein, Free State, South Africa. Sugarcane bagasse (SB) was sourced from Tongaat hullet, Tongaat, Kwazulu Natal, South Africa. Halloysite nanoclay powder (HS), beige-coloured with a formula weight of 294.19 g/mol and a pore size of 1.26–1.34 mL/mol, was purchased from Sigma-Aldrich, Johannesburg, South Africa. Its particles have a diameter of 30–70 nm and a length of 1–3 microns. Expandable graphite (EG) was supplied by Qingdao Kropfmuehl Graphite in Hauzenberg, Germany.

### 2.2. Sample Preparation

#### 2.2.1. Preparation of Sugarcane Bagasse (SB) Fibres

The crushed sugarcane bagasse waste was washed with water using a modified version of a method prescribed by Jalalah et al. [28]. The bagasse was cleaned with distilled water to remove soil, dirt, and other foreign debris. The fibres were then cut into about 2 cm lengths and underwent boiling water treatment for 60 min to remove colouring matter and sugar traces, and this process was repeated three times. The clean fibres were strained to remove excess water and dried at 20 °C for 72 h. After drying, the fibres were pulverised using a powder pulverising machine and sieved through a 1.0 mm sieve.

#### 2.2.2. Fabrication of Composites

Before compounding, PBS, SD, SB, HS, and EG were dried in a vacuum oven at 60 °C for over 12 h. Table 1 shows the calculated weight percentages of the different components in each of the investigated samples. The neat PBS, binary, and hybrid composites were melt-mixed using a TE-30 co-rotating twin-screw extruder (Nanjing Only Extrusion Machinery Co., Ltd., Nanjing, China), with the temperatures of the heating zones from the hopper to the die set from 120 to 160 °C. The extrusion speed and feed rate were set at 178.8 rpm and 4.4 kg/hr, respectively. The extrudates were then quenched in water, pelletized, and dried in an oven at 60 °C for 24 h. These dried pellets were injection moulded into various test specimens using an injection moulding machine (ENGEL e-mac50). The injection moulding heating zones were set at 36, 120, 140, 150, and 160 °C. The clamping force, metering, specific back pressure, injection pressure, and injection speed were set at 500 kN, 29 mm, 100 bars, 550 bars, and 100 m/s, respectively. The test specimens were cooled to 22 °C and stored in a ziplock bag.

### 2.3. Sample Characterization

#### 2.3.1. Microscopic Analysis (Scanning Electron Microscopy—SEM)

Qualitative analysis of the morphological and textural features of the neat polymer, composites, and hybrids was carried out using scanning electron microscopy (SEM). The specimens were initially fractured using liquid nitrogen and then broken into appropriate sizes to fit into the sample chamber in the SEM. After that, they were coated with a thin layer of gold to improve conductivity and imaging quality. These gold-coated samples were then examined using a JSM-7800F Extreme-resolution Analytical Field Emission Scanning Electron Microscope, which was operated at an accelerating voltage of 5.0 kV. To ensure stability and precise imaging, the samples were carefully mounted on 10 mm Cambridge pin-type aluminium stubs using epoxy glue. The high-resolution capabilities of the JSM-7800F allowed for detailed qualitative analysis of the surface morphology and texture of the materials.

#### 2.3.2. Chemical Structures (Fourier Transform Infrared Spectroscopy—FTIR)

Attenuated total reflectance-Fourier transform infrared (ATR-FTIR) spectroscopy (Perkin-Elmer Spectrum 100, PerkinElmer, Branford, CT, USA) was used to determine the chemical structures of PBS, SD, SB, and hybrid PBS/SD/SB/EG/HS. The samples underwent 32 scans at wavelengths ranging from 500–4000 cm^−1^ at a resolution of 4 cm^−1^.

#### 2.3.3. Flow Dynamics (Rheology)

To evaluate the flow and deformation of the fabricated composites under stress, which aids in optimizing manufacturing processes, a modular compact rheometer (Anton Paar) was employed (MCR 302). The tests were conducted using a 25 mm diameter parallel-plates configuration at 180 °C under atmospheric conditions. Preliminary experiments were executed to establish the linear viscoelastic region of the samples, and a fixed strain amplitude of 1.00% was used. The zero gap was maintained at 1.1 mm for all tests. Replicate experiments were carried out to ensure reproducibility.

#### 2.3.4. Thermal Stability (Thermo-Gravimetric Analysis—TGA)

TGA analyses were carried out using a TG analyser (model Q500, TA Instruments, New Castle, DE, USA) to determine the purity and decomposition temperature of the evaluated samples. Specimens with masses ranging between 8 and 10 mg were heated in triplicate at a heating rate of 10 °C/min from 25 to 800 °C under nitrogen. The resulting measurements from the computer-controlled thermo-gravimetric analyser were presented as a curve in which the mass or mass percentage is plotted against the temperature and/or time. Furthermore, a differential thermogravimetric curve DTG was generated as the first derivative of the weight with respect to temperature.

#### 2.3.5. Differential Scanning Calorimetry (DSC)

DSC tests were conducted using a Perkin Elmer DSC7 instrument. Samples with masses ranging between 5 and 6 mg were sealed in aluminium pans. The samples were heated under a nitrogen flow of 20 mL/min from −35 to 160 °C at a heating rate of 10 °C/min and kept at this temperature for 1 min to eliminate the thermal history, then cooled to −35 °C at the same rate and reheated under the same conditions. The melting enthalpies and temperatures were determined from the second heating curves.

#### 2.3.6. Impact Resilience (Charpy Impact Test)

The injection moulded specimens of the different formulations with dimensions of approximately 80 mm × 10 mm × 4 mm (L × W × B) were subjected to a Charpy impact test to evaluate their resistance to breakage by flexural shock. The specimens were notched on one side, with a 0.25 mm notch root radius at a depth of 2 mm, using a CEAST Automatic Notchvis Plus (Instron, Norwood, MA, USA). The notched Charpy impact strength was measured at 25 °C using a fully automated CEAST Pendulum Resil Impactor II (Instron). The drop velocity was 3.7 m/s, resulting in a hammer energy of 14 J. The reported results represent the average of at least six tests per sample.

#### 2.3.7. Mechanical: Tensile

Tensile testing was carried out on the injection moulded, dog bone-shaped specimens using an Instron 5966 tester (Instron Engineering Corporation, Norwood, MA, USA, ASTM 638D). The tensile tester was equipped with a 10 kN load cell, and the tests were conducted at a constant strain rate of 50 mm/min and a temperature of 25 °C. The results are based on an average of six tests per sample, along with standard deviations.

## 3. Results

### 3.1. Scanning Electron Microscopy

**Figure 1:**
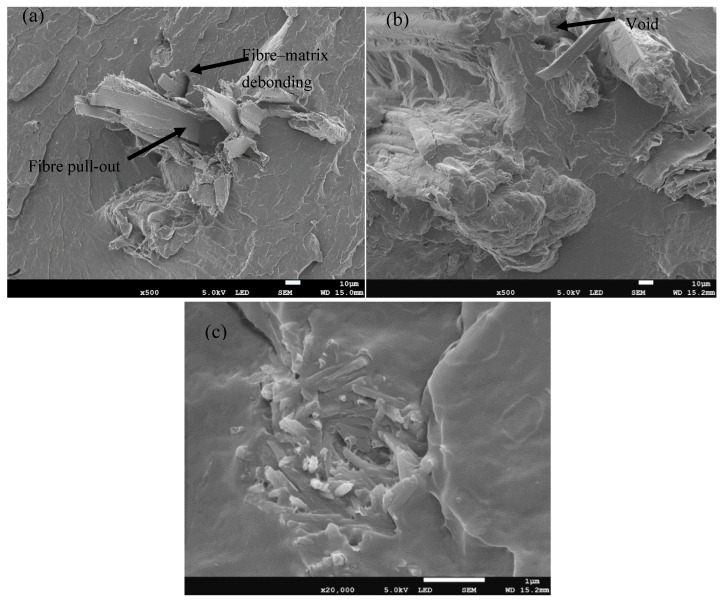
SEM micrographs of binary composites: (**a**) sawdust (SD), (**b**) sugarcane bagasse (SB), and (**c**) halloysite (HS).

**Figure 2:**
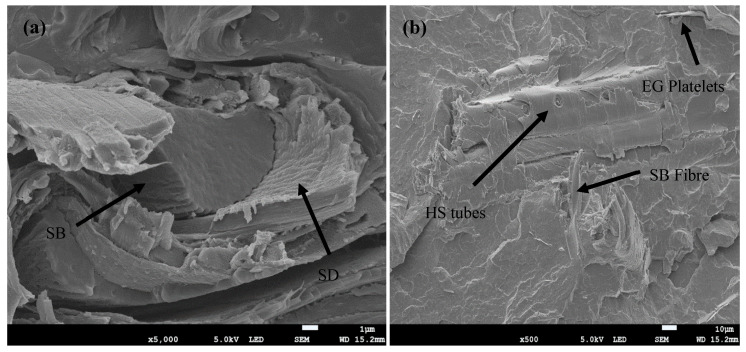
SEM micrographs of hybrid composites: (**a**) PBS/SD/SB and (**b**) PBS/SD/SB/HS/EG.

Figure 1a–c represent the SEM micrographs of the morphologies of the studied composites. The presence of SD in the smooth PBS matrix is characterized by light, flaky, and smooth-looking structures, while the SB fibre is characterized by dark-coloured, coarse, and long parallel-striped structures [29,30]. When these individual fibres, SD and SB, are separately added into the PBS matrix, SB and SD show visible agglomerates, voids, and fibre pull-outs in both composites. The voids and fibre pull-outs can be attributed to the fact that there were no fibre pre-treatments prior to processing. Pre-treatments are crucial for improving the morphology and overall performance of fibre-reinforced polymer composites [31]. The presence of fibre pull-outs and voids on the surface of the composites is typically a result of inadequate adhesion between the fibres and the polymer matrix. This weak interfacial bonding is often due to the lack of chemical compatibility and mechanical interlocking between the fibres and the matrix. Pre-treatments help improve this adhesion by cleaning the fibre surfaces, introducing functional groups that enhance the chemical compatibility with the matrix in the process [31,32]. The simultaneous incorporation of both SD and SB fibres into the PBS matrix resulted in the separation of the fibres within the matrix, with SD characterized by flaky structures and SB symbolized by dark-coloured, coarse, and long parallel-striped structures, as shown in Figure 2a. Interestingly, the interaction between the fibres shows SD concealing or encapsulating SB. When mixed into a polymer matrix, the finer SD particles can settle into the interstices of the larger bagasse fibres, effectively “covering” them. This encapsulation might improve the overall distribution and interfacial bonding within the matrix [33]. According to Figure 1c, the tubular halloysite clay seems to have become fairly embedded into the PBS matrix. Furthermore, there are no obvious cavities, which may suggest that there is a good interfacial interaction between the HS and the PBS matrix, even though there are few visible agglomerates. It is well known that halloysite clay is capable of dispersing more easily in polymer matrices than platy clay such as montmorillonite and kaolin, without any exfoliation [34].

**Figure 3:**
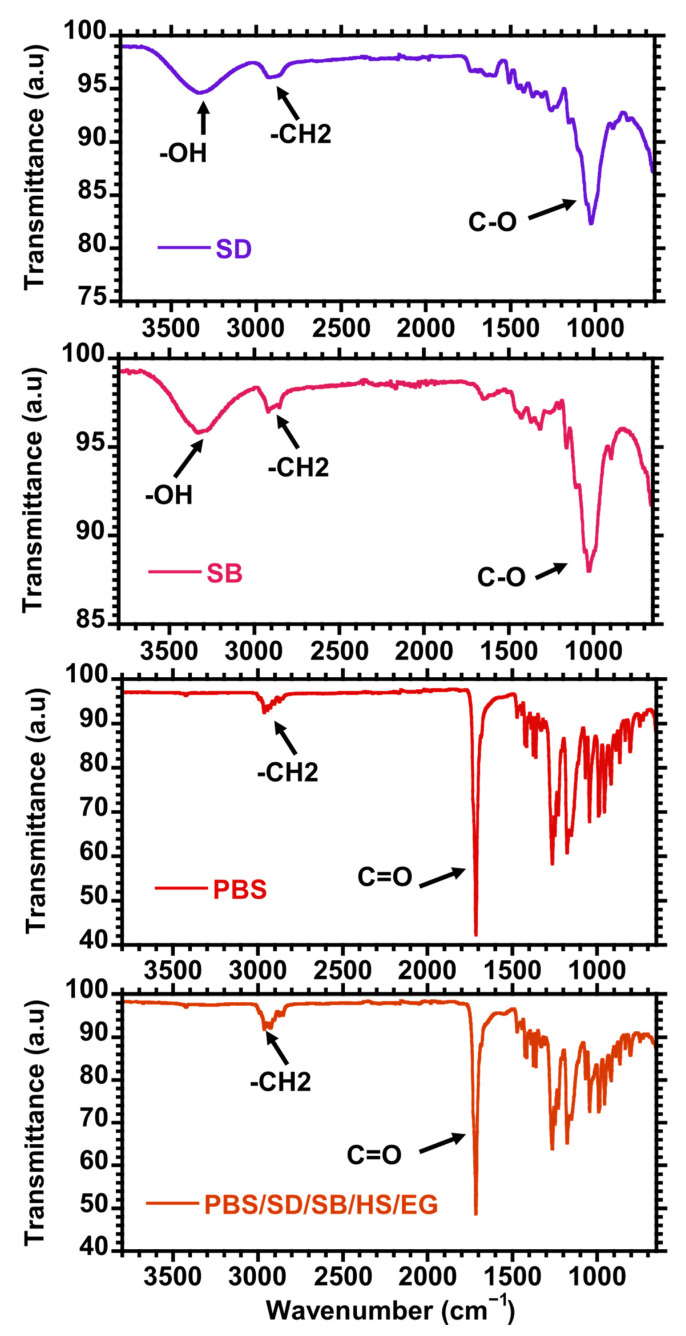
FTIR spectra of PBS, SD, SB, and hybrid PBS/SD/SB/HS/EG composites.

Further improvements in the morphology are observed with the incorporation of HS and EG fillers. Like many mineral clays, HS improves the mechanical interlocking and provides additional surface area for interaction with the polymer matrix, resulting in better dispersion of the fibres and improved adhesion within the matrix. The presence of EG assists in evenly distributing the fibres within the matrix, thereby enhancing the interfacial bonding and reducing the formation of voids and defects. Generally, better dispersion and distribution in the presence of HS and EG can be attributed to the nature of their structures. HS, with its tubular structure, provides a network that supports the distribution of fibres, while EG, due to its layered structure, aids in uniform dispersion and acts as a barrier to fibre agglomeration [35,36,37]. Using polymer composites with better dispersion of filler material(s) usually results in the enhancement of properties such as mechanical performance due to the effective stress transfer provided by the incorporated filler. Thus, in the presence of EG and HS, the PBS/SD/SB hybrid composite could be expected to demonstrate better performance, provided that there is also sufficient interfacial adhesion between PBS and the fillers. The FTIR spectra of PBS, SD, SB, and hybrid composites comprising all the fillers including HS and EG are illustrated in Figure 3. SD and SB are characterized by a broad hydroxyl group (–OH) between 3600 and 3200 cm^−1^ and -CH_2_ stretching at 2950–2800 cm^−1^. Intense C–O stretching vibrations were noticed at 1200–1000 cm^−1^. On the other hand, PBS showed a strong carbonyl vibration at around 1700 cm^−1^. The hybrid composite demonstrated a similar structure to PBS. The vibration bands associated with both SD and SB are not visible in the hybrid composite, and this could be due to the finely dispersed fillers in the continuous PBS matrix.

### 3.2. Rheological Properties of Investigated Samples

**Figure 4:**
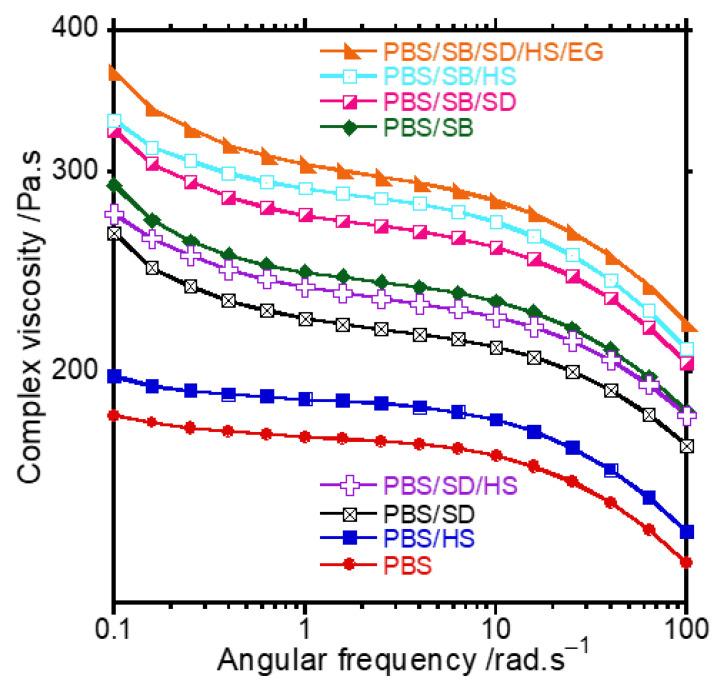
Complex viscosity curves as function of angular frequency of neat PBS, hybrid composites, and ternary composites.

Figure 4 illustrates the complex viscosity (η*) of neat PBS, binary composites, and hybrid composites as a function of angular frequency (ω). PBS generally exhibits a lower η* due to fewer restrictions on chain mobility, allowing it to flow more easily under shear forces [38]. A continuous trend emerges with the addition of different fibres and fillers (EG and HS). Natural fibres are stiff materials and restrict polymer chain mobility, which increases η* [39]. There is, however, a slight difference in the behaviour of the fibres. SB-containing composites display a higher η* than SD composites due to differences in their stiffness within the PBS matrix. It seems as if SD is less stiff when compared with SB, with SB showing greater stiffness in the matrix than SD, and this could be associated with factors such as differences in the chemical compositions, sizes, and aspect ratios of the two fibres. This superiority of SB results in a more significant restriction of polymer chain movement, thereby increasing the η* more than SD. The hybrid mixture of PBS, SB, and SD shows a higher η* than the single-fibre composites, as expected. The positive synergy between the fibres leads to the enhanced stiffness of the fibres within the matrix, thus restricting the PBS chains with more than one fibre. Lastly, the formulation with the HS and EG fibres exhibits the highest η* among all the samples, indicating further stiffening and limited movement of the polymer chains.

### 3.3. Thermal Stability of Composites and Hybrid Composite

**Figure 5:**
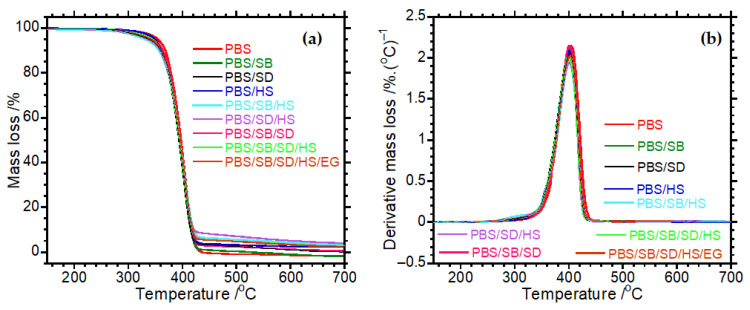
(**a**) TGA curves and (**b**) derivative curves of neat PBS, hybrid composites, and ternary composites.

Figure 5 illustrates typical TGA curves of neat PBS and binary and hybrid composites. The thermal stability of the materials associated with temperatures at 5% (T_5%_) and 50% (T_50%_) mass loss is reported in Table 2. The curves depict weight loss as a function of temperature, offering insights into the thermal stability and degradation behaviour of the samples [40]. The classic one-step degradation curves were observed for all samples, indicating a single dominant degradation process for each material. Incorporating different fillers for either binary or hybrid components did not seem to alter the primary degradation pathway of PBS, as indicated by the similarity in the curves [41,42]. However, the T_5%_ and T_50%_ of the binary and hybrid systems occurred earlier than those of the neat polymer. This early initiation of thermal degradation suggests a potential interaction between the components, which could influence the thermal stability. The shift in onset temperature suggests that the binary and hybrid systems may have different degradation mechanisms or pathways compared to PBS. Additionally, the earlier offset temperature in these systems implies that the degradation process completes more rapidly. This behaviour could be attributed to the presence of additional phases or interfaces in the binary and hybrid systems that catalyse the degradation process at lower temperatures, leading to a reduction in thermal stability. During early decomposition in TGA, test materials tend to release volatile compounds that may affect the degradation mechanism of the materials [42]. For instance, natural fibres release volatiles such as water vapour, acetic acid, and other organic acids [43]. In the case of halloysite, alkali ammonium compounds, including ammonium hydroxide and alkyl ammonium salts, are released during degradation [44]. Expanded graphite, on the other hand, releases volatile acids such as sulphuric acid and phosphoric acid during decomposition [45]. These compounds act as catalysts, accelerating the decomposition process and influencing the formation of the char layer. This, in turn, affects the thermal stability and flammability of the composites. Understanding the role of these volatiles is crucial for designing materials with improved thermal and fire-resistant properties [42,43,44,45].

### 3.4. Differential Scanning Calorimetry

**Figure 6:**
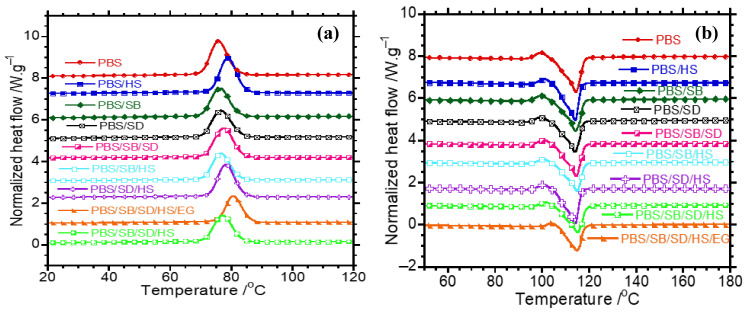
DSC thermograms of (**a**) cooling curves and (**b**) second heating curves of neat PBS, binary composites, and hybrid composites.

Table 3 summarizes the DSC properties obtained during cooling and second heating scans. The corresponding cooling and second heating curves are displayed in Figure 6. PBS has a T_c_ of 75.7 °C. Upon the addition of either SB or SD, the T_c_ remained in the same range as in PBS. A very slight shift was noticed in PBS/SD/SB hybrid composites (77.6 °C), though this might be statistically insignificant compared to single-fibre composites. A notable shift in T_c_ (around 78 °C) was observed in PBS/HS and PBS/SD/HS, which implies the nucleation efficacy of HS in accelerating the crystallization rate of PBS. Horiuchi et al. [6] observed an increased crystallization rate of PBS in PBS/Canabarva fibres/lignin hybrid composites in the presence of MMT and SEP clays, and they attributed this to induced nucleation caused by the clays. This indicates the tendency of nanoclays to facilitate heterogeneous nucleation in polymer matrices. The incorporation of EG into PBS/SD/SB/HS composites showed a huge shift in T_c_ to higher temperatures (~81 °C), insinuating that better heterogeneous nucleation was provided by EG particles.

The melting temperatures of the prepared samples are summarized in Table 3. PBS is a semicrystalline polymer characterized by a melt/recrystallization/re-melt phenomenon [12,46]. Depending on the heating rate and crystallization temperatures, PBS can display multiple melting peaks resulting from different crystal forms, partial melt/recrystallization/re-melting, and so forth [46]. In this case, PBS displayed a small cold-crystallization peak, and a shoulder prior its main melting temperature (T_m_). Modulated DSC studies are important for understanding this complex melting behaviour displayed by PBS. Nevertheless, it is discernible that the T_m_ of PBS did not practically change when the fillers were incorporated, as observed elsewhere [6,11,12]. This observation could mean that the fillers did not have a significant influence on the crystal size of PBS [8]. The melting enthalpies decreased with the addition of the fillers, which implied a reduction in the crystallinity of PBS. This indicates a disruption to chain folding and stacking in the presence of fillers. A reduction in crystallinity has detrimental effects on the stiffness of polymer materials, which subsequently influences the heat resistance properties such as heat distortion temperature (HDT). However, in this case, the fibres and other fillers (HS and EG) compensate the stiffness of PBS despite the reduction in crystallinity.

### 3.5. Dynamic Mechanical Analysis of Composites and Hybrid Composites

**Figure 7:**
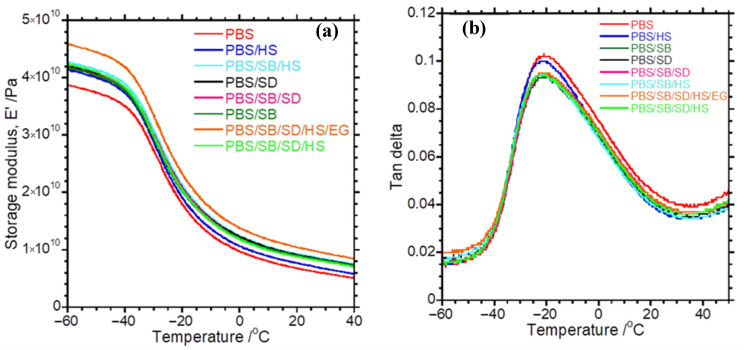
DMA (**a**) storage modulus and (**b**) tan delta.

The viscoelastic properties of the prepared composites were investigated using DMA, which is an important analytical tool to determine a material’s properties, including the stiffness, elasticity, dampening, crosslinking density, etc. The influence of natural fibres on the stiffness of the polymer matrix can be determined from the storage modulus curves in DMA. Plots of the storage moduli and tan delta against the temperature are depicted in Figure 7. In the glassy state, the macromolecular chains are frozen and tightly packed, resulting in the high storage modulus of the polymer. The storage modulus decreases when the temperature is increased due to an increase in chain mobility, making the materials less stiff. It can be observed that PBS exhibited the lowest storage modulus (Figure 7a). Upon the addition of HS, the storage modulus of PBS increased due to the nano-reinforcement provided by clay nanotubes, stiffening the macromolecular chains of the matrix. The incorporation of either SD or SB fibres also increased the storage modulus of PBS, implying the reinforcing effect of the fibres. However, there are insignificant differences in the storage moduli of PBS/SD and PBS/SB composites. The hybrid composite (PBS/SD/SB) showed a similar storage modulus to those of PBS/SB and PBS/SD. This contradicts reported studies that demonstrated enhancement of the storage moduli of hybrid composite systems compared to their respective single-fibre composites [8]. However, this observation could mean that the effect of a single fibre (either SB or SD) on the storage modulus of PBS can be attained by simultaneous incorporation of SD and SB with 50% loading of each fibre. In terms of product development, this suggests that both SD and SB can be beneficiated as reinforcing fillers by the simultaneous incorporation of them at equal volumes in the PBS matrix to generate hybrid composites, instead of developing single-fibre composites at higher concentrations.

The incorporation of HS in either PBS/SB or PBS/SD/SB did not have a significant influence on the storage moduli of these composites, although there was a slight increase in the storage modulus of PBS/SB. The addition of EG into the PBS/SD/SB/HS hybrid composite system showed a remarkable increase in the storage modulus of PBS over the entire range of temperatures. This insinuates that EG has better reinforcing capacity as it has improved the stiffness of the hybrid PBS/SD/SB/HS composite. EG, as a micro-filler, has a tendency to restrict the mobility of macromolecular chains and increase the stiffness of polymer matrices [47,48]. Similar findings have been reported whereby carbon-based materials such as reduced graphene oxide, graphene nanoplatelets, and carbon nanotubes improved the stiffness of hybrid natural fibre/polymer composites [8]. Upon increasing the temperature above the T_g_, the storage moduli of the samples decreased dramatically due to increases in the mobility of PBS chains. At ambient temperatures, however, the EG hybrid system showed the highest storage modulus, suggesting enhanced stiffness at normal service temperatures.

Tan delta plots of all the prepared systems are depicted in Figure 7b. All the samples are characterized by a similar T_g_, which indicates a lack of interfacial adhesion between the fibres and the PBS matrix. Usually, a good interfacial adhesion between the polymer matrix and the fibres increases the T_g_ due to the confinement of the polymer chains by a network of fibres, as observed elsewhere [8]. However, a reduction in the tan delta peak is noticed in the composites, implying increased dampening of PBS.

### 3.6. Mechanical Properties of Composites and Hybrid Composites

**Figure 8:**
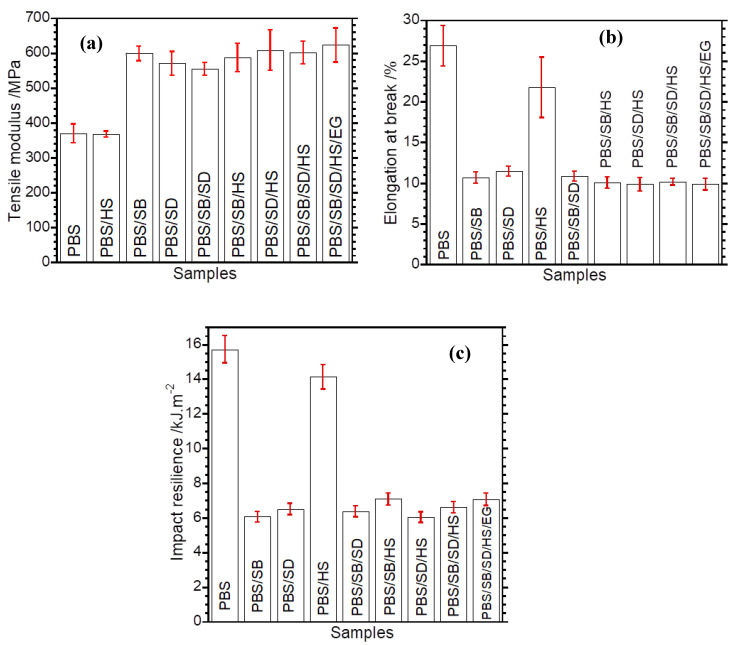
(**a**) Tensile modulus, (**b**) elongation at break, and (**c**) impact resilience.

One of limiting factors for using PBS is its ductility, which poses a challenge where stiffness is required. Generally, stiff materials such as natural fibres could compensate for low stiffness in soft biopolymers such as PBS. The tensile modulus, which provides information regarding the stiffness of PBS and its respective composites, is shown in Figure 8a. PBS and PBS/HS are characterized by a low tensile modulus of approximately 380 MPa. The fibre-reinforced composites showed a significant increase in tensile modulus, which is in accordance with the observed increase in storage modulus in DMA (Figure 7a). The PBS/SB/SD hybrid composite showed a slightly lower tensile modulus compared to the single-fibre composites, while the hybrid composite containing HS and EG showed the highest tensile modulus, reaching above 600 MPa. On the other hand, the elongation at break, which relates to the toughness of a material, confirmed the ductility of PBS, as it showed the highest elongation at break. However, a huge reduction in elongation at break was noticed upon the incorporation of the fibres, as displayed in Figure 8b. The hybrid composites containing HS and EG showed an elongation at break in a similar range. The elongation at break has a strong dependence on matrix–fibre adhesion for effective stress transfer. In this case, the morphology analysis indicted a lack of compatibility, which explains the reduction in the toughness. Usually, a reduction in elongation at break is accompanied by a decrease in impact resistance.

The impact resilience of the prepared samples is shown in Figure 8c. PBS showed the highest resistance to impact, due to its ductile nature and ability to absorb energy during fracture. A sudden decrease in impact resilience can be noticed in all the composites. The observed decrease could be attributed to the increased rigidity and stiffness of PBS upon the incorporation of the fibres. Usually, stiff polymer materials tend to show low impact resistance. Further, the lack of interfacial adhesion between the fibres and the PBS matrix contributed to insufficient stress transfer from the PBS matrix to the fibres. Better compatibility in polymer-reinforced fibre composites allows efficient stress transfer from the polymer matrix to the fibres, thus increasing the energy required to fracture the material. The impact resilience of all the hybrid composites was below 50% of that of neat PBS. The observed trend indicates the need to functionalize the fibres prior to blending with the polymer matrix to allow efficient stress transfer during impacting while maximizing the energy absorption capacity of the material.

## 4. Discussion

In this study, the influences of natural fibres (SB and SD) on the material properties of PBS prepared through the melt compounding technique were investigated. The influences of HS and EG on the properties of the respective PBS/SB/SD hybrids were also evaluated. To elucidate the effects of individual fibres, single-fibre composites of PBS/SD and PBS/SB were also prepared. The morphological analysis showed fibre pull-outs and voids in both PBS/SD and PBS/SB composites. These observations indicated poor interfacial compatibility between the fibres and the PBS matrix, which has a huge amount of potential to adversely impact the mechanical performance. On the other hand, the hybrid PBS/SD/SB composite system demonstrated the encapsulation of SB by flaky SD fibres. The rheological characterization indicated an increase in the viscosity of PBS/SD/SB compared to PBS/SD and PBS/SB, indicating the synergy of the fibres in improving the flow viscosity of PBS. One of the challenges of using PBS is its low melt viscosity and melt strength. Increasing the viscosity of polymers could indicate, in some instances, the enhancement of melt strength, which is essential for processes such as foaming. In this study, the incorporation of HS also resulted in the enhancement of the melt viscosity of single-fibre composites, with EG-containing hybrid composites showing the greatest improvements. Though the melt viscosity increased with fibre inclusion, all the composites could still be extruded without altering the processing conditions used to prepare neat PBS. The thermal stability of all the composites decreased, and this is attributed to the additional phases released from the fillers that catalysed the degradation process of PBS. Although the onset temperature decreased in PBS/SB compared to PBS, the hybridization with SD (PBS/SD/SB) led to an increase in this temperature, with a further slight increase for the PBS/SD/SB/HS and PBS/SD/SB/HS/EG composites. The DSC analysis confirmed increases in the crystallization temperatures of PBS and its respective hybrid composites in the presence of HS and EG. The presence of EG showed better heterogeneous nucleation in PBS/SD/SB/HS hybrid composites, indicating that EG promoted chain folding and stacking of PBS chain segments and the formation of crystals at the matrix–filler interfaces. Faster crystallization rates are industrially important for processes such as injection moulding, where the parts of items can be manufactured within short cycle times.

**Figure 9:**
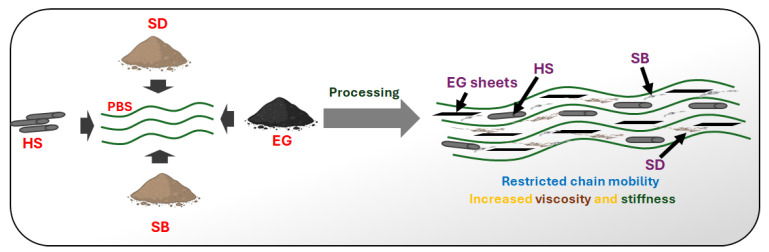
Scheme for reinforcing PBS with SB, SD, HS, and EG.

The thermomechanical measurements indicated an increased storage modulus of PBS when the fibres were added. The maximum improvement in this property was noticed when EG was included in the PBS/SBS/SD/HS composite. At a normal service temperature (~ 23–25 °C) this composite indicated enhanced stiffness, responding to our aim to increase the stiffness of PBS as a viable way to expand its applications. Increasing the stiffness has a direct influence on other properties such as HDT. The tensile modulus also improved after adding the fibres (binary and hybrid composites), insinuating the reinforcing effect of the added fillers on restricting the mobility of PBS chains, as shown in Figure 9. The reduction in both toughness and impact could be attributed to a lack of interfacial compatibility between the fibres and the PBS matrix, implicating the need to pre-treat the fibres or incorporate compatibilizers such as PBS grafted with moieties like maleic anhydride. This will bring a balance in mechanical properties such as stiffness and toughness. Overall, the prepared hybrid composites show great potential in developing green composites for applications such as rigid packaging, cutlery, and many others. Future studies should dwell on improving the interfacial adhesion between the fibres used and PBS and evaluate the environmental impact of the prepared composites at the end-of-life stage.

## 5. Conclusions

Herein, the effects of the natural fibres SB and SD on the properties of PBS prepared through melt compounding were investigated. The influences of the incorporation of HS and EG on the properties of PBS/SB/SD hybrid composites were also examined. The morphological analysis indicated poor interfacial adhesion between PBS and the fibres. The obtained findings indicated enhancements in the complex viscosity of PBS in the presence of natural fibres, and further improvements in the presence of HS and EG. Moreover, the crystallization temperatures of PBS increased in the presence of fillers, with EG showing better nucleation efficiency. The stiffness of PBS/SD/SB composites also increased upon the addition of HS and EG. However, the toughness and impact resilience decreased due to the increased stiffness of the composites and possibly due to poor interfacial adhesion between the matrix and the fillers, indicating the need to pre-treat the fibres to enhance compatibility. Overall, the prepared hybrid composites could be utilized to develop rigid materials for various purposes such as packaging.

## Figures and Tables

**Table 1:** Weight percentages of components of composites.

Samples	Percentages (wt.%)
PBS	100
PBS/HS	97/3
PBS/SD	90/10
PBS/SB	90/10
PBS/SB/SD	90/5/5
PBS/SB/HS	87.3/9.7/3
PBS/SD/HS	87.3/9.7/3
PBS/SB/SD/HS	87.3/4.85/4.85/3
PBS/SB/SD/HS/EG	87.3/4.85/4.85/1.5/1.5

**Table 2:** Summarized onset (T_5%_) and offset (T_50%_) degradation temperatures.

Samples	T_5%_/℃	T_50%_/℃
PBS	356.7	400
PBS/SB	329.4	395
PBS/SD	338.1	396.1
PBS/HS	348.6	394.4
PBS/SB/HS	336.7	396.7
PBS/SD/HS	337.8	396.7
PBS/SB/SD	337.5	395
PBS/SB/SD/HS	341.1	395.6
PBS/SB/SD/HS/EG	340.0	395.6

T_5%_ and T_50%_ represent the decomposition temperature at 5% and 50%, respectively.

**Table 3:** Summarized DSC properties of prepared samples.

Sample	T_c_ (°C)	T_m_ (°C)	ΔH_m_ (J/g)
PBS	75.7	114.4	71.4
PBS/SB	76.6	114.2	63.5
PBS/SD	76.6	114.0	60.6
PBS/HS	78.9	114.1	65.6
PBS/SB/HS	76.5	115.0	57.2
PBS/SD/HS	78.2	114.0	64.0
PBS/SB/SD	77.6	114.5	63.1
PBS/SB/SD/HS	77.2	115.2	56.6
PBS/SB/SD/HS/EG	80.6	115.0	48.3

## Data Availability

The data are contained within the article.
